# Boron in Diet and Medicine: Mechanisms of Delivery and Detection

**DOI:** 10.3390/ph19010081

**Published:** 2025-12-30

**Authors:** Dorota Bartusik-Aebisher, Izabela Rudy, Kacper Rogóż, David Aebisher, Gabriela Henrykowska

**Affiliations:** 1 Department of Biochemistry and General Chemistry, Collegium Medicum, Faculty of Medicine, University of Rzeszów, 35-959 Rzeszów, Poland; dbartusikaebisher@ur.edu.pl; 2 English Division Science Club, Collegium Medicum, Faculty of Medicine, University of Rzeszów, 35-310 Rzeszów, Poland; akacja03@interia.pl (I.R.); kr117626@stud.ur.edu.pl (K.R.); 3 Department of Photomedicine and Physical Chemistry, Collegium Medicum, Faculty of Medicine, University of Rzeszów, 35-959 Rzeszów, Poland; 4 Department of Epidemiology and Public Health, Faculty of Medicine, Medical University of Lodz, 90-419 Lodz, Poland

**Keywords:** boron, isotopes, nanocarriers, BNCT

## Abstract

Boron is a trace element with multifaceted chemical and biological properties that underpin its emerging relevance in human health and medicinal chemistry. Although present in organisms at very low concentrations, boron participates in key physiological processes, including mineral metabolism, bone homeostasis, hormonal regulation, immune modulation, and redox balance. Its unique electronic structure—characterized by electron deficiency and the ability to form multi-center bonds—gives rise to diverse allotropic, cluster, and coordination chemistries, enabling the formation of biologically active complexes and therapeutic agents. Dietary boron, derived mainly from plant-based foods, is efficiently absorbed and predominantly excreted by the kidneys, showing a strong correlation between intake and urinary levels. Current evidence suggests beneficial effects of boron on bone mineral density, cognitive function, inflammation, antioxidant defenses, and metabolic regulation, although the precise molecular mechanisms remain partially understood. In medicinal chemistry, a broad spectrum of boron-containing compounds—including borates, boronic acids, boronated amino acids, carboranes, and metallacarboranes—has gained clinical and preclinical importance. These compounds serve as enzyme inhibitors, antimicrobial and anti-inflammatory agents, metabolic modulators, and critical boron carriers in boron neutron capture therapy (BNCT), which leverages the neutron-capture properties of ^10^B for targeted cancer treatment. Advances in synthesis, functionalization, and nanocarrier design have expanded the therapeutic potential of boron-based molecules. Ongoing research aims to optimize their selectivity, biodistribution, safety, and diagnostic integration. Overall, boron represents a versatile and rapidly developing component of modern biomedical science, with promising implications for oncology, infectious diseases, metabolic disorders, and precision medicine.

## 1. Introduction

### 1.1. The Importance of Trace Elements in Biology and Medicine

Trace elements are micronutrients that occur in organisms at very low concentrations but perform key biological functions as enzymatic cofactors, cell signal modulators, and structural components of proteins. Their balance is essential for human health, as deficiency or excess can disrupt metabolic processes, affect the immune system, tissue development, and organ function. Particularly for bone tissue, trace elements such as boron influence calcium and phosphorus metabolism and regulate osteoblast and osteoclast activity, which is important in the prevention of osteoporosis and other bone diseases [1,2].

### 1.2. Chemical and Physical Properties of Boron

Boron (B) is an unusual element from group 13 of the periodic table—it possesses characteristics of both a nonmetal and a semimetal, which translates into its complex chemistry. In nature, it occurs primarily in the form of borates (e.g., boraxes, boric acid), rather than in its elemental form. Boron has the ability to form coordination bonds through empty p-orbitals, allowing the formation of complex structures such as borate esters with diols (e.g., sugars), which is crucial for its biological interactions. Furthermore, borates exhibit a high affinity for hydroxyl groups, enabling the formation of molecular bridges and affecting the stability of biological structures. Physically, boron typically occurs in biological conditions as borate ions or boric acid, which influences its bioavailability and transport within organisms [3]. Boron is characterized by electron deficiencies, a wide range of allotropic structures, and unusual oxidation states. In nature, boron occurs mainly in two stable isotopes: ^10^B (~19–20%) and ^11^B (~80–80.9%) [4,5]. The ^11^B/^10^B ratio is variable in different geochemical environments and used in hydrological and environmental studies [5]. These isotopes are important not only for chemistry but also for technology—for example, ^10^B has a very high neutron absorption cross section, making it crucial in nuclear applications (e.g., in BNCT) [6]. Furthermore, mechanical studies have shown an “anomalous isotope effect” in thin films such as boron or boron nitride monolayers—differences in the nuclear charge distribution between ^10^B and ^11^B can affect the mechanical propertcies of the material [7]. The most common oxidation state of boron in compounds is +3—it predominates in oxides, halides, borates, and many other compounds [6,8]. However, boron can also assume unusual, lower oxidation states. For example, in some boron compounds or clusters, the +2 state or lower is possible. Studies indicate that the reduction from B(III) to B(II) can be initiated in nontrivial ways—for example, by dehydrocoupling borates with iodine [9]. Furthermore, modern theoretical (and experimental) analyses suggest that in specific clusters or nanomaterial structures, boron can exhibit highly complex electronic chemistry and multi-site bonding (e.g., three-site 3c–2e bonds) [10]. Boron forms a wide variety of allotropic structures, which results from its low number of valence electrons and ability to form multi-site bonds [11,12]. One of the most classic images is the icosahedral B_12_ cluster (icosahedron)—these 12-atom units combine linto form larger structures in crystalline boron (e.g., α- and β-boron) [12,13]. The high-pressure boron phase, known as γ-B_28_, contains B_12_ clusters (icosahedrons) and dumbbells (B_2_) in a structure resembling the NaCl-type network, with partial charge transfer between cluster elements, indicating partial ionicity of bonds [14]. In the field of boron nanomaterials, boron clusters of dimensions 0D (e.g., cage-like “borospheres”), 1D (nanotubes), and 2D (borophenes) have been observed [11]. Moreover, boron clusters can function as building blocks in polymers, for example, icosahedral borates used in hybrid polymers, where they can form both classical 2c–2e and 3c–2e bonds, giving them unique steric and electronic properties [10]. Electronic rules such as Jemmis’ rule (mno rules) help understand the electronic arrangement and structure formation of these complex boron clusters, linking theory with their bonding properties [15].

### 1.3. The Role of Boron in Plant and Animal Organisms

In plants, boron is widely considered an essential element: it stabilizes cell walls through esterification with pectins, regulates ion transport, and influences meristem growth. Its deficiency leads to tissue deformation, inhibited root growth, and deterioration of cell wall structure, while excess boron can be toxic, indicating a narrow range of homeostasis [16]. In animal organisms, including humans, boron appears to play bioactive roles, although it is not always classified as an essential element. Studies indicate its influence on calcium metabolism, bone mineral density, steroid hormone levels (e.g., estrogen, testosterone), as well as immunological and metabolic functions [17]. Furthermore, boron can interact with cell membranes and influence cell physicochemistry, which may be important in its mechanism of action [18].

### 1.4. Overview of Current Research on Boron in the Context of Human Health

In recent years, interest in the role of boron in human health has been growing rapidly. Literature reviews indicate that boron supplementation may positively affect bone mineral density by modulating calcium, vitamin D, and sex hormone metabolism [19]. Key experimental animal studies have shown that boron supplementation improves bone strength and microstructure (e.g., in diabetic mice [20] and in rats subjected to intense exercise [21]). Furthermore, population-based and interventional studies in humans have shown that doses of approximately 3 mg/day of boron support bone health and reduce urinary calcium excretion, without exceeding safe intake limits. However, mechanistic gaps remain: for example, the precise effects of boron on cell signaling and the long-term effects of supplementation are not fully understood. Therefore, further clinical and basic research is needed to better define the optimal doses, safety range, and mechanisms of action of boron in humans [19]. It is important to note that while animal models provide strong mechanistic data (e.g., on embryogenesis), human evidence is largely derived from cross-sectional epidemiological studies and small-scale interventions, necessitating cautious extrapolation of these findings to clinical practice.

**Figure 1:**
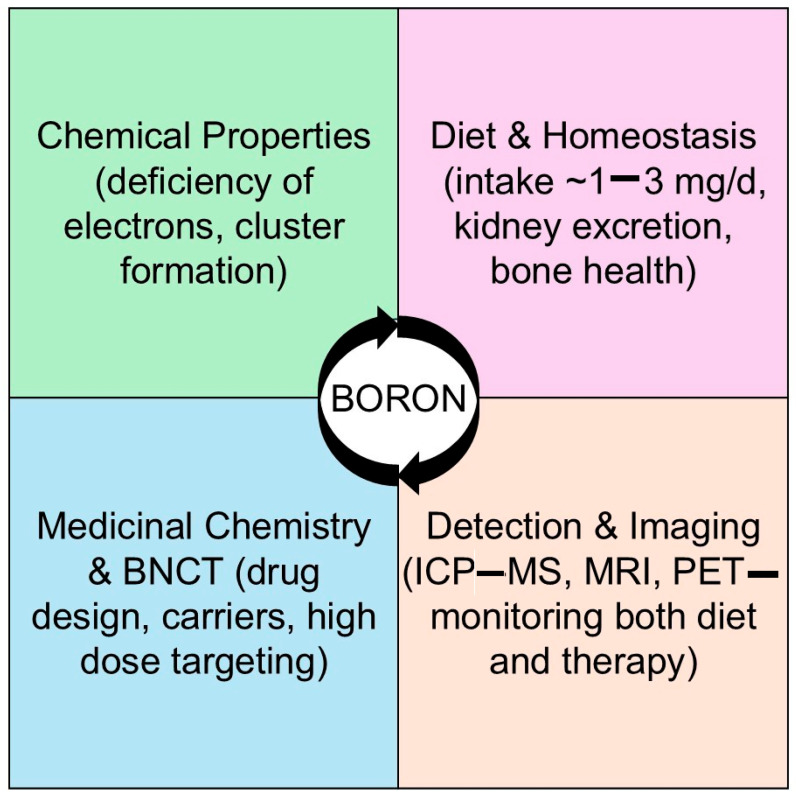
Integrative conceptual framework of the review (created by the authors).

Unlike previous reviews that focus solely on nutritional aspects or exclusively on BNCT chemistry, this article aims to present an integrated view of boron. We posit that the successful development of advanced boron therapies requires a deep understanding of its fundamental dietary homeostasis and metabolic fate. By connecting the dots between basic biological handling of dietary boron, modern synthetic capabilities, and sophisticated delivery and detection systems, we provide a holistic roadmap for leveraging boron’s unique properties in precision medicine (Figure 1).

### 1.5. Literature Search Methodology

This paper was designed as a narrative review aimed at synthesizing interdisciplinary knowledge regarding boron’s role in nutrition, medicine, and chemical synthesis. A comprehensive literature search was conducted using electronic databases, including PubMed, Web of Science, Scopus, and Google Scholar. The search strategy prioritized articles published between 2000 and 2024 to capture recent advancements, although seminal historical papers describing fundamental properties of boron were also included. The search was performed using combinations of the following keywords: “boron essentiality,” “dietary boron,” “boron toxicity,” “BNCT therapy,” “boron carriers,” “boronic acids in drug design,” “Suzuki-Miyaura coupling,” and “boron nanomaterials.” The selection criteria focused on peer-reviewed original research, meta-analyses, and clinical trials published in English. Articles were screened for relevance to three core pillars: human physiology, medicinal chemistry applications, and synthetic methodologies, with a particular emphasis on identifying connections between chemical structure and biological activity.

## 2. Boron Content and Metabolism in the Human Diet

### 2.1. Dietary Sources of Boron

Boron (B) is found primarily in plant foods; the richest sources are dried fruits (e.g., raisins, prunes), nuts, seeds, leafy vegetables, and legumes. Many analytical studies have shown that boron concentrations in fruits and vegetables are significantly higher than in most animal products or cereal products, and drying fruit concentrates this element (significantly higher in dried vs. fresh fruits). The average daily boron intake in studied Western populations typically ranges from approximately 0.5 to 3 mg/d, with diets rich in fruits, vegetables, nuts, and legumes yielding higher intakes [22,23,24,25]. Drinking water and beverages (including wines and fruit juices) also contribute to boron intake: the boron content of water and beverages varies geographically and may constitute a significant proportion of the total intake in some populations. Moreover, products with a high volume of consumption (e.g., coffee, milk) may be significant “contributors” to the total intake despite the relatively low boron concentration in a single portion [25,26]. The boron content in food (Table 1) depends on the type and part of the plant, soil conditions, processing and analytical method, consequently, the values from food composition tables may differ between countries and publications [22,23,24].

### 2.2. Boron Absorption, Metabolism, and Excretion

Boron in food and water occurs primarily as soluble anions (boric acid/borates), which are readily and rapidly absorbed intestinally in humans. Data from intervention studies and urinary excretion measurements indicate high bioavailability of dietary boron and a strong correlation between intake and urinary concentration [26,30]. After absorption, boron circulates in the blood primarily as the undissociated form (boric acid) or complexes with low-molecular-weight ligands; it is not extensively metabolized by enzymatic pathways, meaning that most of the ingested boron remains soluble and is exchanged between body fluids and tissues. Some tissues (e.g., bone, nails) exhibit a higher tendency to accumulate boron, but a significant portion of the ingested boron is eliminated within a short time (days) [27,28,30]. The primary route of elimination is the urinary system—measurements of daily urinary boron excretion are a commonly used biomarker of intake and exposure. Intervention studies demonstrate that after oral supplementation, a significant portion of the dose (e.g., ~80–85% of the dose after 10 mg B/d for 4 weeks) is rapidly recovered in the urine, indicating high absorption and effective renal excretion. In renal impairment, boron clearance is reduced, which may lead to accumulation [26,29,30]. Boron influences the metabolism of other minerals (e.g., calcium, magnesium, phosphorus) and steroid hormone concentrations; its physiological effects and fate in the body are partially modulated by these metabolic relationships [27,28,30].

### 2.3. Biological Functions of Boron

#### 2.3.1. Role in Calcium, Magnesium, and Vitamin D Metabolism

Boron (B) plays a significant role in mineral homeostasis and vitamin D metabolism. Studies suggest that boron intake is associated with increased calcium and magnesium retention in the body and influences vitamin D activity. One review indicates that boron influences the activity of 25-hydroxyvitamin D and may modify calcium metabolism by influencing parathyroid hormone and calcitonin secretion [28]. Furthermore, experimental data show that boron-deficient diets in animals lead to poorer bone mineralization, suggesting boron’s involvement in osteogenesis and bone mass maintenance [31].

In a seminal metabolic ward study by Nielsen et al., boron supplementation (3 mg/day) in postmenopausal women reduced urinary calcium excretion by 44% and urinary magnesium excretion, while significantly elevating serum 17β-estradiol concentrations. These quantitative findings suggest that boron may function by interacting with steroid hormone metabolism or extending the half-life of Vitamin D and estrogen, rather than acting solely on the mineral matrix [32].

#### 2.3.2. Impact on Bone Health, Cognitive Function, and the Immune System

In the context of bone health, boron intake has been correlated with improved bone mineral density and a reduced risk of osteoporosis, especially with diets rich in other minerals (calcium, magnesium) [33]. Regarding cognitive function, there is experimental and observational data that low boron status may be associated with impaired cognitive performance and neurological function [31]. Regarding the immune system, boron and boron compounds have been shown to modulate both innate and adaptive immune responses. For example, boron-containing compounds influence the production of pro-inflammatory cytokines and T cell function, suggesting a potential role for boron in regulating inflammation and immunity [34].

#### 2.3.3. Potential Anti-Inflammatory and Antioxidant Properties

A growing body of evidence suggests that boron may possess anti-inflammatory and antioxidant properties. A literature review found that diets with higher boron intake were associated with lower concentrations of inflammatory markers (such as CRP) and a more efficient antioxidant response [35]. Experimental studies have shown that in wound and tissue injury models, boron accelerated healing, which is partially attributed to its ability to modulate free radical production and inhibit excessive inflammatory responses. Although the mechanisms of action are not fully understood, possible pathways include effects on cyclooxygenase, cytokine production, and antioxidant enzyme activity [36].

#### 2.3.4. The Role of Boron in Embryonic Development

The role of boron in embryogenesis provides some of the strongest evidence for its essentiality in the animal kingdom. Pioneer studies using the African clawed frog (*Xenopus laevis*) model demonstrated that boron deficiency during early development leads to severe morphological defects, including abnormal gastrulation, necrotic changes, and organ malformations. It was observed that low boron levels impair the assembly of the cytoskeleton and cell-to-cell adhesion, which are critical for proper tissue morphogenesis [37].

A breakthrough in understanding this mechanism was the identification of the mammalian electrogenic borate transporter, NaBC1 (encoded by the *SLC4A11* gene). This transporter, which functions as a Na^+^-coupled borate cotransporter, is essential for maintaining intracellular boron homeostasis and is highly expressed in developing tissues. In zebrafish (Danio rerio) models, boron deficiency or the knockdown of the boron transporter resulted in high embryonic mortality and photoreceptor degeneration [17].

Mechanistically, recent research suggests that boron interacts with the Wnt/β-catenin signaling pathway, a highly conserved pathway regulating cell proliferation and differentiation during embryogenesis. Boron appears to influence the expression of Wnt ligands and the stability of β-catenin. Consequently, optimal boron levels are required for the correct execution of developmental programs, while both deficiency and excess can disrupt these delicate signaling cascades, leading to teratogenic effects (e.g., skeletal and cardiovascular malformations) observed in toxicological studies at high doses [38,39].

Table 2 summarizes the biological effects of boron and the proposed mechanism of its action.

### 2.4. Toxicity and Recommended Daily Intake

#### 2.4.1. Safe Intake Range (UL, ADI)

Although boron has not been formally recognized by many organizations as an essential element for humans (there is no official RDA), certain safe intake limits have been established (Table 3). According to a literature review, the tolerable upper intake level (UL) for adults has been established at approximately 20 mg/day in the USA/Canada. In Europe, the European Food Safety Authority (EFSA) has established an acceptable daily intake (ADI) of ~0.16 mg/kg body weight, which for a 70 kg person corresponds to approximately 11.2 mg/day [40,41]. In practice, typical intake is approximately 1–3 mg/day [40].

#### 2.4.2. Symptoms of Deficiency and Excess

**Figure 2:**
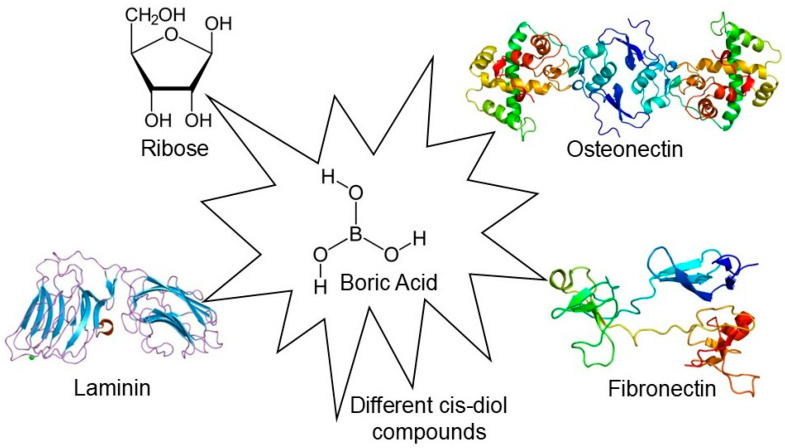
Mechanism of serine protease inhibition by boronic acids. The formation of a reversible covalent bond between the boron atom and the serine hydroxyl group mimics the tetrahedral transition state, effectively blocking enzyme activity (created by the authors).

Mechanisms of Boron Deficiency: Although specific enzymes requiring boron as a strictly essential cofactor in humans have not been fully identified, the mechanistic effects of boron deficiency are linked to its affinity for cis-diol groups (Figure 2). One proposed mechanism involves the interaction of boron with S-adenosylmethionine (SAM). Animal studies suggest that boron deprivation increases SAM utilization and elevates homocysteine levels, thereby disrupting methylation pathways crucial for DNA synthesis and gene expression. Furthermore, boron is hypothesized to play a structural role in cell membranes by cross-linking glycoproteins and glycolipids containing cis-diol residues. Its deficiency may impair membrane integrity and the function of transmembrane proteins (e.g., ion channels). Additionally, low boron status has been associated with the upregulation of inflammatory pathways; normally, boron helps suppress NF-κB activity, so its absence leads to increased levels of inflammatory cytokines like TNF-α and IL-6, potentially explaining the negative impact of deficiency on bone and brain health [31,35,43,44].

Mechanisms of Boron Toxicity: Boron toxicity, typically resulting from excess boric acid intake, involves different molecular targets. A primary mechanism of acute toxicity is the inhibition of crucial enzyme systems. High concentrations of borates can bind to the hydroxyl groups of active sites in serine proteases and other metabolic enzymes, mimicking the transition state and blocking catalytic activity. Another significant mechanism is the depletion of cellular riboflavin (vitamin B2). Boric acid forms complexes with riboflavin, leading to its increased urinary excretion and subsequent metabolic disturbances. In terms of reproductive toxicity—the most sensitive endpoint in animal studies—excess boron specifically targets Sertoli cells in the testes. The mechanism involves the disruption of DNA synthesis and inhibition of histone deacetylases (HDACs), leading to germ cell apoptosis and testicular atrophy. At the systemic level, severe acute toxicity can induce metabolic acidosis and renal failure due to the accumulation of boric acid, which exceeds the kidney’s clearance capacity [41,45].

Understanding the natural homeostatic mechanisms and safety limits of dietary boron is crucial when designing therapeutic agents. Since the body possesses efficient clearance pathways (renal excretion), developing boron-based drugs requires strategies to either utilize these pathways or modify the chemical structure to ensure adequate retention time at the target site. This transition from nutritional intake to pharmacological application is explored in the following section.

## 3. Overview of Boron Compounds of Therapeutic Importance

### 3.1. Boron Compounds

Boron-containing compounds (in forms such as borates, boric acids and their derivatives, boronated amino acids, carboranes, and boron clusters/metallocarboranes) have gained increasing importance in medicinal chemistry over the past two decades. They are used both directly as pharmaceuticals (e.g., boron-core protease inhibitors), as boron carriers for neutron capture therapy (BNCT), and as building blocks for imaging and drug delivery molecules. General reviews and synthetic articles describe the growing interest in these classes of compounds and their diverse therapeutic and diagnostic applications. [46,47].

Polyboron anions (e.g., dodecaborane, B12-clusters) and their derivatives exhibit unique physicochemical properties: a high boron content in a small volume, relatively low acute toxicity, and the potential for easy surface functionalization. As a result, they are intensively studied as boron carriers for BNCT and as fragments enhancing lipophilicity/biostability in anticancer and antibacterial drugs. Recent studies also indicate the potential of dodecaborates and clusters in combination therapy (e.g., with small-molecule drugs or nanocarriers) [48,49].

The class of boronic acids (including compounds such as benzoxaborole, boronates, and trifluoroborates) is one of the most active in drug design. The characteristic ability to form covalent, reversible bonds with hydroxyl and oxygen/nitrogen-containing groups (e.g., serine and threonine residues in enzymes) enables the creation of potent enzyme inhibitors—examples of clinical success include proteasome inhibitors and other compounds targeting enzymes with a hydroxyl-containing active moiety. Medicinal chemistry reviews discuss the design of boronate-containing drugs, along with their successes and challenges (stability, specificity, pharmacokinetics) [46,50,51].

Boronated amino acids (e.g., L-p-boronophenylalanine—BPA and various cyclic and unnatural amino acids with a boron substituent) have been and are being intensively studied as boron carriers for BNCT. Their advantages include often selective uptake by rapidly dividing tumors (amino acid transporters, e.g., LAT1) and the ability to introduce a large number of boron atoms into tumor cells. Experimental studies and clinical reviews analyze the synthesis, biodistribution, and efficacy of various boronated amino acids, as well as strategies for improving selectivity and tumor retention [52,53,54].

Carboranes (Cage-like C–B–H structures, e.g., closo-carboranes) are unique, sterically compact, and hydrophobic pharmacophores that can replace phenyl, t-butyl, or other aromatic moieties in drug design, providing unique lipid-binding properties and metabolic resistance. Carboranes have been used both as elements enhancing ligand binding to proteins (e.g., in enzyme inhibitors) and as boron-rich carriers for BNCT. Reviews and experimental studies demonstrate their application in the design of anticancer and anti-inflammatory drugs, as well as in biological chemistry [55,56,57].

Metallacarboranes (boron-metallic compounds) and other cluster anions (with favorable electronic properties and geometry) demonstrate promising antibacterial, antiviral, and anticancer activities in preclinical models. Their specific properties—stability, surface modifiability, and electrostatic interactions—make them interesting candidates for further pharmacological studies. Review papers from recent years have highlighted the growing interest in these structures and discuss translational challenges [6,58].

Boron compounds constitute a multifaceted class of chemotherapeutic agents and therapeutic carriers—from small boric acids and boronic enzyme inhibitors, through boronated amino acids (BNCT carriers), to carboranes and metallacarboranes with unique steric and electronic properties. The increasing pace of research, new reviews and experimental work indicate real clinical potential in targeted anticancer therapy, BNCT and other diagnostic and therapeutic applications (Table 4) [46,47,55].

### 3.2. Clinical Applications

#### 3.2.1. Boron Neutron Capture Therapy (BNCT)—Cancer Therapy

**Figure 3:**
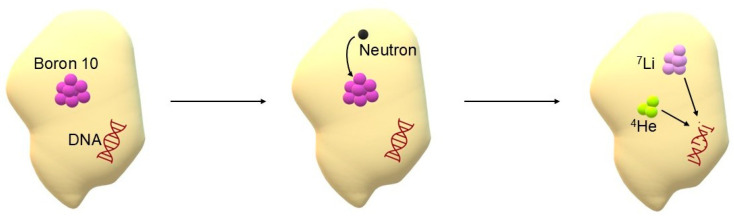
Schematic representation of the boron neutron capture therapy (BNCT) mechanism. The capture of a thermal neutron by the Boron 10 (^10^B) nucleus results in the release of high-energy α particles (^4^He) and ^7^Li ions, causing localized lethality to cancer cells (Created by the authors).

Boron neutron capture therapy (BNCT) is a promising radiotherapeutic modality utilizing the ^10^B isotope, which, upon neutron capture, emits high-energy particles (α particles and Li ions), destroying cancer cells with minimal damage to healthy tissue [58]. This local and selective therapy method is particularly attractive for tumors located in areas difficult to treat with traditional radiotherapy. Clinically used boron carriers include p-boronophenylalanine (BPA) and BSH (sodium borocaptate), although their limitations (e.g., insufficient selectivity, leaching from the tumor) motivate the development of new, multifunctional carriers. Research on modern boron (>^10^B) delivery agents focuses on molecules such as peptides, liposomes, nanoparticles, and porphyrins, which are modified to increase specific uptake in tumor cells and enable imaging of boron distribution before and during therapy [59]. The theranostic approach to BNCT is also gaining increasing interest: combining a boron carrier with an imaging component (e.g., a fluorophore, a PET tracer), which allows for noninvasive determination of ^10^B concentration in the tumor and surrounding tissues and optimization of neutron dose planning. However, a key challenge in BNCT remains accurate monitoring of boron distribution in tissues. Currently, ^10^B measurements in tissues are limited—they are often based on the assumption of a fixed ratio of uptake from plasma to tissue, which in reality can be subject to significant error. Development of new boron delivery platforms that combine therapy with imaging and proteomics (e.g., detection of the expression of proteins associated with boron transport) could significantly improve the precision and efficacy of BNCT in clinical use (Figure 3) [60].

A major milestone in the clinical translation of BNCT was achieved in 2020 in Japan. The Japanese regulatory agency (PMDA) approved the world’s first accelerator-based BNCT system (NeuCure™) combined with a borofalan ^10^B drug product (Steboronine™) for the treatment of unresectable, locally advanced, or recurrent carcinoma of the head and neck. This approval marked the transition of BNCT from experimental nuclear reactor sites to hospital-based accelerator centers. Currently, accredited centers actively treating patients or finalizing commissioning are predominantly located in Japan, including the Southern Tohoku BNCT Research Center and the Kansai BNCT Medical Center (Osaka Medical and Pharmaceutical University) [61]. Outside of Japan, significant progress is being made in Helsinki, Finland (Helsinki University Hospital) and in Xiamen, China, where accelerator-based facilities are entering clinical phases [62,63].

Regarding clinical efficacy, recent trials have demonstrated promising results. The pivotal Phase II study (JHN002) in Japan involving patients with recurrent head and neck carcinoma showed an overall response rate (ORR) of 71.4%, with a one-year survival rate of approximately 94%, which is significantly higher than standard therapies for this patient group [64]. Clinical trials for glioblastoma (GBM) and malignant melanoma are also ongoing, with results suggesting a survival benefit and an improved safety profile compared to conventional radiotherapy, primarily due to the high cellular selectivity of the heavy ion irradiation [65,66].

#### 3.2.2. Applications in the Treatment of Infections, Inflammation, and Metabolic Diseases

Boron compounds are not limited to cancer therapy. In the context of metabolic diseases (such as diabetes, lipid disorders), it has been demonstrated that natural and synthetic boron compounds can affect enzymes involved in carbohydrate and lipid metabolism and modulate membrane transporters—suggesting their therapeutic potential in the treatment of metabolic disorders in humans [67]. Furthermore, boron compounds, especially boric acids and derivatives (e.g., boronate and boronate esters), exhibit anti-inflammatory and antibacterial effects. Boron inhibitors of bacterial enzymes and modulators of inflammatory processes have been described in the literature, raising the prospect of using these compounds as anti-inflammatory or antibacterial drugs [68].

#### 3.2.3. New Research Directions: Boron in Anticancer, Antiviral, and Antibacterial Drugs

Modern medicinal chemistry is increasingly exploring single boron atoms (e.g., in boronates, benzoxaboroles) as part of therapeutic drugs. Reviews indicate that boron’s unique properties—the ability to form reversible covalent bonds with amino acids or hydroxyl groups—can be exploited to design enzyme inhibitors in cancer, viral, or bacterial cells [69].

In the antiviral and antibacterial fields, boron protease inhibitors (e.g., viral proteases, β-lactamases) and other mechanisms of action are being investigated. Furthermore, boron carborane structures, metallacarboranes, and other boron clusters are being modified to optimize antimicrobial activity and pharmacokinetic properties [68,69]. There is also growing interest in boron pro-drugs and drug carriers that can combine therapy with molecular targeting and imaging, opening new avenues in oncology, infections and precision medicine [70].

### 3.3. Mechanisms of Action

#### 3.3.1. Interaction with Enzymes and Ion Channels

Boron-containing compounds, particularly boronic acids and boronate derivatives, exhibit a characteristic enzyme inhibitor mechanism involving the formation of reversible, covalent complexes with nucleophilic residues in the active site of enzymes—most often serine or threonine hydroxyls. Boron ligand binding changes the boron atom’s hybridization from sp^2^ to sp^3^, mimicking the tetrahedral transition state of the substrate hydrolysis reaction and leading to potent inhibition. This mechanism underlies the clinical success of boron-based inhibitors (e.g., bortezomib, a proteasome inhibitor) and numerous new drug designs targeting proteases, β-lactamases, and other drug-metabolizing enzymes [71,72]. In addition to classical hydrolytic enzymes, modernly designed boron compounds (e.g., β-aminoboronates, arylborates) are used as selective inhibitors of enzymes involved in lipid metabolism and cell signaling. Examples from activity-based profiling show that boron chemotypes can provide highly selective, cell-active inhibitors of enzymes (an example being the inhibition of (ox)lipid-metabolizing enzymes) [71,73]. Importantly, certain boron fragments also function as pharmacophores for ion channels and transmembrane receptors. A classic example is the compound 2-aminoethoxy-diphenylborate (2-APB), used experimentally as a modulator of calcium storage channels and various TRP subtypes. 2-APB exhibits complex, concentration- and channel-type-dependent effects: blockade of some TRPC/TRPM channels and activation of others (e.g., TRPV1–3), which demonstrates that the boron moiety can confer unique pharmacological profiles to ligands relative to ion channels [74,75,76].

#### 3.3.2. The Role of Boron in Modulating Oxidative Stress

A growing body of evidence from in vitro studies, preclinical studies, and reviews indicates that boron (and some of its compounds) has the ability to modulate cellular redox balance. Mechanisms described in the literature include the following: (i) influence on antioxidant enzyme activity (e.g., increased superoxide dismutase (SOD) activity, catalase) and glutathione levels in experimental models, (ii) reduction in lipid peroxidation markers, (iii) modulation of the inflammatory response by reducing proinflammatory mediators, and (iv) stimulation of regenerative processes (cell proliferation and migration in wound healing models). These effects are form- and dose-dependent—there is a therapeutic window during which boron exerts a protective effect; outside this window, excess boron can be toxic. Studies on the use of boron in wound healing and tissue protection suggest that the antioxidant and antibacterial properties of boron work together to accelerate wound closure and reduce the risk of infection. Additionally, some metabolic models have demonstrated an effect of boron on enzymes involved in glucose and lipid homeostasis, paving the way for research into its use in metabolic diseases (although the molecular mechanisms require further clarification) [28,36].

#### 3.3.3. Mechanisms of Selective Uptake in Cancer Tissues (e.g., in BNCT)

The most well-known example of selective uptake is p-boronophenylalanine (BPA)—a carrier used in BNCT. Transport studies have shown that LAT1 (L-type amino acid transporter 1) is the primary determinant of BPA uptake at clinically used concentrations, while at higher concentrations, the ATB(0,+) transporter also becomes important. LAT1 is often overexpressed in many tumor types (high amino acid demand in tumors), which explains the preferential accumulation of BPA in cancer tissue and is one of the pillars of BPA-based BNCT selectivity [77]. Another mechanism is the physicochemical properties of the carrier: large boron clusters (carboranes, dodecaborates), liposomes, polymeric nanocarriers, or porphyrin conjugates are designed to utilize the EPR (enhanced permeability and retention) effect in tumors—through increased tumor vascular permeability and insufficient lymphatic drainage. Additional surface functionalization (targeting peptides, antibodies, ligands) improves recognition and retention in tumors, thus increasing the T/N (tumor/normal) ratio [59]. In BNCT, the mere accumulation of significant amounts of ^10^B in the tumor is not sufficient—the production of α and ^7^Li particles has a range of several micrometers, so the maximum biological effect is achieved when boron atoms are close to critical targets (e.g., DNA or organelles). Therefore, carriers are designed to penetrate cells and preferentially localize in cellular compartments of cytotoxic importance [35,65]. Effective BNCT requires accurate measurement of ^10^B concentrations in tumor and normal tissues during treatment planning and before irradiation. Analysis methods include chemical techniques (e.g., ICP-MS), neutron spectroscopy, PET methods (for theranostic conjugated carriers), and emerging boron imaging techniques. Limitations in the sensitivity, specificity, and availability of measurement methods, as well as pharmacokinetic variability in patients, remain major translational barriers [59,78]. Current directions of development include the design of theranostic carriers (combining the ^10^B delivery function with an imaging component), peptide and antibody conjugates (to increase selectivity), and the use of metallocluster structures (e.g., metallacarboranes) to combine pharmacological properties with high boron content. Initial work with boron nanocarriers and carboranes has shown promising antitumor effects in preclinical models but requires further safety and efficacy studies [48,59].

## 4. Synthesis of Boron Compounds

The diverse therapeutic applications described above—ranging from small-molecule enzyme inhibitors to complex BNCT carriers—are only possible due to advancements in synthetic chemistry. The ability to precisely install boron atoms into organic scaffolds and create stable clusters underpins the entire field of boron medicinal chemistry.

### 4.1. Classical Methods

Classical methods for the synthesis of boron compounds encompass a number of well-established procedures. Boric acid (H_3_BO_3_) is typically produced industrially by reacting boron oxide (B_2_O_3_) with water or by hydrolysis of boron esters; laboratory-scale preparation by hydrolysis of trialkyl boron esters is also possible. Borohydrides (e.g., NaBH_4_) are synthesized by reduction of boron esters or by reactions of metallohydrides with haloborates; detailed modifications of the procedures also allow the preparation of complex transition metal borohydrides or anhydrous organoborane derivatives. In recent years, more efficient and scalable methods for preparing various types of boranes have been developed (including NaBH_4_ activation procedures for the synthesis of amine- and phosphine-borates), which facilitate the preparation of borane complexes and their further functionalization [79,80].

Condensation reactions (e.g., the formation of borane esters by condensing B(OR)_3_ with diols, amines, or hydroxyls) are not merely of academic interest but are fundamental to drug manufacturing. For example, the final steps in the synthesis of peptide boronic acid drugs like Bortezomib often involve the precise protection and condensation of the boronic acid moiety to ensure stability and activity [81,82].

### 4.2. Modern Synthesis

Virtually all modern strategies for introducing boron fragments into molecules are based on borylation reactions (C–B generation) and cross-linking reactions, in which boron compounds act as donors for the formation of C–C or C–heteroatom bonds. Suzuki–Miyaura (Pd-catalyzed) remains the most versatile and widely used method for combining organoborones with halogen derivatives, enabling the construction of aryl-aryl and aryl-alkyl bonds and applications in the synthesis of complex pharmaceutical systems. Mechanistic and technological advances (e.g., the use of trifluoroborates, MIDA-boronates, photocatalysis, Ni catalysts for difficult partners) have significantly expanded the range of substrates and reaction conditions [83,84].

The Chan–Lam/Chan–Evans–Lam (copper) reaction is an effective method for formtransitioning C–N and C–O bonds using arylboronates/boronates under mild conditions, often without the need for strong bases or rigorously anaerobic conditions. This reaction has become particularly useful in the synthesis of biologically active compounds (arylation of amines, amides, phenols), and numerous reviews summarize catalyst developments and extensions of the reaction scope [85].

Modern direct borylation methods (e.g., Ir-catalyzed C–H borylation) enable the direct insertion of the B group onto the C–H positions of heteroarenes. This strategy is invaluable for late-stage functionalization, allowing medicinal chemists to directly install a boron ‘warhead’ into complex, pre-existing drug scaffolds. This approach significantly accelerates the discovery of novel benzoxaboroles (such as Tavaborole analogs) and facilitates the rapid preparation of molecular libraries for structure-activity relationship (SAR) studies [86].

In addition to metal-catalyzed processes, numerous metal-free borylation methods (e.g., activated diboron reacting with alkenes/alkynes, borene electrophiles, photochemical and radical methods) have been developed over the past decade, offering greater compatibility with sensitive functionalities and improved sustainability. Examples include photoinduced borylation of haloarenes and metal-free deoxygenative borylations of alcohols—these methods expand the synthetic repertoire of organoborons with a smaller metallic footprint [87,88].

Towards sustainable synthesis, adaptations of classical and new reactions to “green chemistry” conditions are being pursued: aqueous solvents or aqueous-organic mixtures, reactive borylations at low temperatures (photochemistry), continuous flows (flow chemistry), and waste minimization. Low-metal catalysts and alternative energy sources (light, electrochemistry) are increasingly being used. Simultaneously, work is emerging on biocatalytic methods for introducing boron fragments (enzymes modified to form C–B bonds)—a field still in its early stages, but offering the potential for high selectivity and a “green” process profile [82,89].

The design of “borono-biological” compounds (drugs containing boron fragments, peptide/antibody conjugates with boron groups, carboranes and metallacarboranes as carriers) combines chemical synthesis strategies with the tools of medicinal chemistry. In practice, modular approaches are used to synthesize BNCT carriers like p-boronophenylalanine (BPA) derivatives or antibody-drug conjugates. This involves: (i) synthesis and protection of boron fragments (e.g., boronates, boronate esters), (ii) conjugation with biological molecules (chemoselective conjugation), (iii) final functionalization to a biologically compatible form (prodrugs, targeting groups). Such molecules are often designed with theranostics in mind (combining therapeutic capacity with imaging capability) and as ^10^B carriers for BNCT—hence the growing demand for syntheses enabling the rapid preparation of multifunctional boron conjugates [81,82].

## 5. Delivery Systems for Boron Compounds

### 5.1. Challenges Related to Bioavailability and Selectivity

#### 5.1.1. Penetration Across Biological Barriers (e.g., Blood–Brain Barrier)

One of the key challenges in delivering boron to brain tumors is the blood–brain barrier (BBB). Standard boron carriers (such as BPA) may have limited access to brain tissue, making it difficult to achieve therapeutic concentrations of ^10^B in tumor cells. Therefore, alternative strategies are being developed, such as administering boron compounds into the cerebrospinal fluid (CSF), which bypasses the BBB. In tumor-bearing rat models, administration of BPA via the CSF results in increased accumulation in tumor tissue with relatively low boron levels in healthy tissues [90]. Additionally, reviews indicate the development of boron carriers that can actively penetrate the BBB, including modifying the carrier surface and using a ligand targeting receptors present on brain endothelial cells [91].

#### 5.1.2. Extrasystemic Toxicity

When boron carriers are administered systemically, there is a risk of accumulation in non-targeted tissues, which can lead to toxicity. Although many boron compounds are safe as long as no neutron reaction occurs, long-term retention of carriers (e.g., nanoparticles) in organs such as the liver or spleen may raise concerns. Reviews of nanoparticle systems emphasize the need to design biodegradable structures that can be eliminated from the body after their therapeutic function [92,93].

### 5.2. Modern Delivery Systems

It is critical to distinguish between delivery systems designed for BNCT and those for conventional boron-based drugs. For standard drugs, the goal is simply bioavailability. In contrast, BNCT requires a high specific accumulation of ^10^B within the tumor cells—typically 20–50 µg/g—while maintaining low levels in surrounding healthy tissue [36,78]. The efficacy of a BNCT carrier is often defined by the Tumor-to-Normal (T/N) and Tumor-to-Blood (T/B) ratios. Standard carriers like BPA typically achieve T/N ratios of 2.5–4.0 depending on LAT1 expression [53,77], whereas advanced nanocarriers (e.g., liposomes, dendrimers) aim for ratios exceeding 5.0 to minimize collateral damage and utilize the EPR effect [94,95,96,97,98].

#### 5.2.1. Nanocarriers (Liposomes, Nanoparticles, Dendrimers)

Nanotechnology plays a significant role in modern boron delivery systems. A literature review indicates that boron nanoparticles (e.g., polymeric or lipid carriers) improve intratumoral boron concentration and ^10^B maintenance during BNCT [93,94]. Classical liposomes were designed to encapsulate ionic boron compounds both within an aqueous core and within a phospholipid layer. Animal studies have demonstrated high tumor-to-blood ratios following administration of liposomal formulations containing boron compounds [95,96]. Advanced liposomes (“boronsome”)—modern liposomes based on carborane phospholipids (“boronsome”)—were designed to combine BNCT with chemotherapy and imaging (theranostics). Preclinical studies have shown strong tumor accumulation and significant inhibition of tumor growth after neutron deposition [97]. Dendrimers are used as carriers to accumulate a large number of boron atoms in a single macromolecular system. Carborane-functionalized dendritic structures exhibit promising biological properties and selectivity towards cancer cells in BNCT models [98].

#### 5.2.2. Tumor-Targeted Systems

The design of targeted boron delivery systems aims to increase selectivity and minimize accumulation in healthy tissues. An example is antibody-functionalized nanoparticles: for example, boron carbide particles (B_4_C) conjugated with antibodies specific for tumor receptors (LDLR, EGFR) have recently been developed, which increases intracellular boron uptake in cell lines with high expression of these receptors [99]. In a review of multifunctional boron carriers (containing targeting ligands, fluorophores, or radiolabels), the authors emphasize that this approach can significantly improve boron biodistribution and enable imaging (BNCT treatment planning) and therapy [59].

#### 5.2.3. Boron-Containing Antibody Drug Conjugates (ADCs)

Although still in the early stages of development, concepts for antibody-boron conjugates exist. An example is an in silico-designed antibody (so-called Boron Delivery Antibody, BDA), in which specific amino acids of the antibody have been replaced with boronated residues (e.g., carboranes) in a way that does not impair antigen binding. Such BDA theoretically enables very selective delivery of ^10^B to cancer cells while retaining the properties of the specific antibody [100].

## 6. Detection and Quantification of Boron

Effective delivery of boron to target tissues, particularly in the context of BNCT, is futile without the ability to precisely quantify its accumulation. Therefore, the development of sensitive detection and imaging methods is inextricably linked to the progress in therapeutic delivery systems.

### 6.1. Spectroscopic and Analytical Methods

ICP-MS and ICP-OES are instrumental techniques widely used for the quantitative determination of boron in environmental and biological samples. ICP-MS offers very low detection limits and isotope determination capabilities, but can be sensitive to matrix interferences (especially in high-salt samples), while ICP-OES (ICP-AES) remains a robust method for measurements at higher concentrations and in routine work; comparisons and reference issues between these methods have been extensively discussed in the literature [101,102].

^11^B NMR is a direct, non-invasive method enabling the study of boron chemical species and the monitoring of boron compounds (e.g., BNCT carriers) in vitro and in vivo; ^11^B NMR provides structural and quantitative information but requires relatively high boron concentrations and specialized instrumentation (low sensitivity compared to ICP-MS). Contrast methods and techniques to improve sensitivity (e.g., surface coils, longer acquisitions) and relaxation constraints are described in reviews and experimental studies [103,104].

Fluorescence spectroscopy are fluorescence sensors based on ligands binding boron groups (e.g., boronate/boronic acid-based sensors), which enable cellular imaging and simple detection of boron compounds in biological systems. These techniques are particularly useful for subcellular localization of boron compounds and for rapid screening, although they often require specific probe synthesis and may have limited quantitative accuracy without calibration scaling [105,106].

Ion chromatography and gas chromatography are separation methods used to separate boron species (e.g., borates, borate esters) prior to detection. Modern approaches combine IC with a mass spectrometry detector (IC-ESI-QqQ-MS) to improve selectivity and sensitivity in aqueous samples; classical GC (with appropriate derivatization) enables the determination of some volatile and derivative boron compounds in biological matrices. For challenging matrices, sample preparation procedures (e.g., pyrohydrolysis) are used prior to chromatographic analysis [107,108,109]. Practical considerations for sampling and preparation: boron determination in tissues and fluids requires careful control of impurities, selection of digestion/extraction acids, and consideration of matrix inhibition effects—particularly important at low concentrations (<1 mg/kg). Calibration, use of isotopic standards (ICP-MS), and method validation are critical for reliable results [110,111].

### 6.2. Biological Imaging

PET (Positron Emission Tomography)—enables the assessment of the pharmacokinetics and biodistribution of radiolabeled boron carriers (e.g., ^18^F-FBPA), which is particularly useful in planning BNCT and assessing whether a given carrier delivers sufficient boron to the tumor. The development of “theranostic” carriers (structurally identical therapeutic and diagnostic compounds) improves the ability to predict boron concentrations based on PET studies. PET does not directly measure the boron isotope, but allows for estimation of the carrier’s biodistribution [112,113,114].

MRI/^11^B-MRI—MRI: of ^11^B oxide (i.e., the boron nucleus signal) provides direct imaging of boron, but is limited by the low sensitivity of the ^11^B nucleus and short relaxation times. In practice, ^11^B-MRI is useful for preclinical studies and for assessing large boron accumulations, and work is ongoing to improve sensitivity and use specific probes [103,115].

Neutron imaging/neutron autoradiography and activation techniques—methods that measure the distribution of ^10^B after its neutron activation (e.g., neutron-activated autoradiography, neutron imaging) provide quantitative images of boron distribution in tissue sections; these techniques were classic tools in BNCT studies for validating local ^10^B concentration. Alpha/neutron autoradiography allowed correlation with tissue morphology but required processing of radioactive samples [116,117].

SIMS (Secondary Ion Mass Spectrometry/ToF-SIMS/dynamic SIMS) is a mass imaging technique with high spatial resolution (even subcellular) used to localize and quantify boron (including ^10^B) in single cells and tissue sections. SIMS enables mapping of boron distribution at the micrometer level and assessment of accumulation in the nucleus or cytoplasm, which is directly relevant to the effectiveness of BNCT. Limitations include sample preparation (freezing, drying), matrix effects, and the need for calibration against standards [118,119,120].

Alternative mass imaging methods (MALDI-MSI, alpha autoradiography) are also gaining importance, enabling the combination of molecular data with tissue morphology and enhancing the reliability of measurements obtained using classical methods [121,122].

For clinical samples, a combination of (i) sample preparation (acid digestion or extraction, contamination control), (ii) quantitative determination by ICP-MS/ICP-OES or ICP-AES for precise values, and (iii) local imaging (SIMS, autoradiography) to assess heterogeneous distribution is typically used. BNCT planning often compares concentrations in plasma, healthy tissue, and tumor, and therapeutic decisions are based on tumor/blood relationships and subcellular localization [78,101,118].

### 6.3. Boron Chemical Sensors and Biosensors

Fluorescent sensors based on boronate units (boronic acids) and chelating ligands are designed to detect boron-containing compounds (e.g., BPA) in fluids and cells; many probes allow for direct intracellular imaging and rapid detection in environmental samples. These sensors are attractive for pre-monitoring and localization, but require quantitative validation against reference methods (ICPs) [105,106,123].

Electrochemical Sensors and Biosensors—Development of electrochemical boron detectors and biosensors using specific ligand-boron reactions or enzymatic systems has shown promising results in detecting boron in environmental samples; these sensors can offer rapid and portable monitoring solutions, but require stability and selectivity in real-world matrices [78].

Applications in BNCT monitoring—integration of imaging techniques (PET with ^18^F-labeled analogues, ^11^B-MRI) with quantitative analytical methods (ICP-MS, SIMS) and benchtop sensors can create a multiparameter system for monitoring boron delivery to the tumor and assessing treatment effectiveness; the development of theranostic carriers and radiolabeled analogues facilitates the translation of diagnostic results into therapeutic decisions (Table 5) [112,113,114,118].

## 7. Challenges and Future Perspectives

### 7.1. Long-Term Human Exposure and Safety

Despite numerous toxicological and epidemiological studies, significant knowledge gaps remain regarding the long-term effects of exposure to various forms of boron in humans—especially at low, chronic exposures and in sensitive groups (pregnant women, individuals with renal failure). Data from toxicological analyses indicate possible reproductive and developmental effects at high doses, but the epidemiological evidence remains inconclusive and limited by the lack of precise exposure measurements.

To resolve these uncertainties, future research must prioritize large-scale, prospective cohort studies that utilize precise biological monitoring (blood/urine concentrations) and strictly control for confounding factors. Meta-analyses and long-term observations are needed to definitively assess the chronic effects of low doses and interactions with other environmental and dietary factors [124,125]. Establishing a clear safety profile is a prerequisite for broader supplementation guidelines [6,124].

### 7.2. Development and Regulation of Boron-Based Therapeutics

Organic boron compounds (boronic acids, benzoxaboroles, and heterocyclic structures) show increasing therapeutic potential—ranging from enzyme inhibitors (e.g., proteasomes) to antibacterial, anti-inflammatory, and anticancer agents. While approved drugs already exist (confirming translational feasibility), the field faces challenges related to the unique chemistry of these molecules.

Detailed studies of pharmacokinetics, metabolism, and toxicity profiles of specific boron forms in the context of long-term administration are necessary [47,68,126]. A parallel priority is to engage regulators and the medical community to develop specific guidelines for the clinical use of these novel chemotypes, integrating data from medicinal chemistry and molecular biology to accelerate translation to the clinic [6,47].

### 7.3. Technological Advances in In Vivo Boron Quantification

One of the key challenges for boron-based therapies (e.g., BNCT) and for exposure monitoring is the development of sensitive, noninvasive methods for in vivo boron quantification. Current approaches include indirect radiological methods (PET with radiolabeled carriers like ^18^F-FBPA), direct spectroscopic assays (^11^B-MRI/^11^B-MRS), and ex vivo validation techniques (neutron imaging). Each has limitations: PET is indirect, while ^11^B-MRI suffers from low sensitivity requiring hardware optimization [127,128,129].

The future lies in hybrid strategies which improve the sensitivity of ^11^B-MRI and develop theranostic carriers (e.g., nanodiscs) that combine therapeutic delivery with high-resolution imaging [67,127]. Furthermore, establishing analytical standards to facilitate the comparability of results between laboratories (e.g., reference standards for calibrating PET/ICP-MS cross-measurements) is urgently needed to facilitate precise dosimetry in clinical trials [6,130].

## 8. Conclusions

Although rarely considered a key dietary component, boron has a multifaceted effect on the human body. Studies indicate that with moderate consumption (approximately 1 mg/day), boron influences hormone metabolism, bone mineralization, and nervous system function, which may translate into health benefits such as improved bone density and cognitive function [31,32,131]. However, there are also warning signs: some epidemiological studies suggest a possible positive association between elevated blood boron levels and an increased risk of all-cause mortality, underscoring the need for further evaluation of the long-term effects of its consumption. For instance, a prospective cohort study from northern Germany (n = 863) observed that while plasma boron concentrations were associated with all-cause mortality in crude models, this association lost statistical significance after multivariable adjustment for confounders (HR: 1.03; 95% CI: 0.99–1.07). Notably, sex-stratified analyses revealed a specific positive association in women (HR: 1.11 per 5-unit increment; 95% CI: 1.03–1.18), whereas no such risk was observed in men. Given the relatively low number of events in these subgroups, these findings should be interpreted with caution, but they underscore the need for further evaluation of long-term boron exposure, particularly in specific demographic groups [132]. The therapeutic prospects for boron are very promising. Combining modern boron chemistry with medicine has led to the development of compounds with anticancer properties and vehicles for boron neutron capture therapy (BNCT) [6,133,134]. The role of theranostic strategies combining therapeutic function with imaging is growing in boron diagnostics and BNCT. For example, radiolabeled boron carriers allow for tracking boron distribution in the body and optimizing the therapeutic dose. Furthermore, further development of quantitative, non-invasive methods for measuring boron in vivo, such as imaging of radiative neutron reaction products or advanced radiological techniques, could significantly improve the precision of BNCT and patient safety monitoring [60,134].

## Figures and Tables

**Table 1:** Boron content in selected food products (mg/100 g).

No.	Product	Boron Content (mg/100 g)	Source
1	Raisins/dried grapes	≈2.2	[27]
2	Dried plums	≈1.2–2.0	[23,27]
3	Avocado (raw)	≈1.2–1.43	[27,28]
4	Nuts (e.g., almonds)	≈1.6–2.8	[28,29]
5	Peanut butter	≈1.45–1.92	[27,28]
6	Peanuts (dry)	≈1.7	[27]
7	Dried fruits (general)	≈1.5–5.4	[23]
8	Legume products (e.g., soybeans)	≈0.7–1.64	[24]
9	Apples (raw)	≈0.28–0.36	[27,29]
10	Grapes/grape juice	≈0.34–0.49	[28,29]

**Table 2:** Summary of the biological effects of boron on specific systems and proposed mechanisms of action.

System/Tissue	Observed Biological Effects	Proposed Mechanisms	References
Bone and Skeletal	Increased bone mineral density (BMD); improved osteogenesis; reduced urinary calcium excretion.	Regulation of gene expression involved in bone growth; interaction with calcium, phosphorus, and magnesium metabolism; modulation of steroid hormones (e.g., estrogen).	[19,20,31]
Central Nervous System	Improvement in cognitive performance, short-term memory, and attention; essential for brain development (animal models).	Modulation of cell membrane permeability; influence on electrophysiological activity; regulation of enzyme activity in the brain.	[31]
Immune System	Modulation of inflammatory response; accelerated wound healing.	Inhibition of serine proteases involved in inflammation; suppression of NF-κB pathway; reduction in oxidative stress markers (ROS scavenging).	[34,35]
Metabolism	Regulation of lipid and carbohydrate metabolism; potential insulin-mimetic effects.	Binding to NAD+ or inhibition of specific metabolic enzymes; interaction with cell membrane receptors affecting nutrient transport.	[20]
Reproductive System	Essential for embryonic development (in lower vertebrates); toxic to testes at high doses.	Interaction with Wnt/β-catenin signaling pathway (development); inhibition of HDACs in Sertoli cells (toxicity).	[17,37,38,39]

**Table 3:** Range of safe boron intake and symptoms of deficiency/excess.

Parameter	Range/Value	Source
Typical Dietary Intake	1–3 mg/day	[40]
ADI (EFSA)	0.16 mg/kg/day (~11 mg/day for a 70 kg body weight)	[41]
UL (USA/Canada)	20 mg/day	[41]
Symptoms of Deficiency	Reduced bone mineral density, cognitive impairment, impaired immunity	[31,35]
Symptoms of Excess	Nausea, vomiting, diarrhea, liver and kidney damage, impaired fertility	[40,41,42]

**Table 4:** Classification of therapeutically important boron compounds, their mechanisms and development challenges.

Compound Class	Primary Application	Mechanism of Action	Key Advantages	Key Challenges	References
Boronic Acids (e.g., Bortezomib)	Oncology (Proteasome inhibition)	Forms reversible covalent bond with serine/threonine hydroxyls in enzymes.	High potency; proven clinical efficacy; reversible binding.	Poor pharmacokinetics; rapid clearance; potential off-target binding.	[46,50,51,52,53,54,55,56,57,58,59,60,61,62,63,64,65,66,67,68,69,70,71]
Benzoxaboroles (e.g., Tavaborole)	Antimicrobial/Anti-inflammatory	Inhibits leucyl-tRNA synthetase or PDE4 enzymes via boron-oxygen bonding.	Good physicochemical stability; broad spectrum of activity.	Development of resistance; membrane permeability issues.	[68,69]
Boronated Amino Acids (e.g., BPA)	BNCT Carrier	Selectively transported via LAT1 into tumor cells.	Exploits metabolic demand of tumors; low systemic toxicity.	Rapid washout from tumor; requires continuous infusion; modest Tumor/Blood ratio (~3:1).	[52,53]
Polyhedral Boranes (e.g., BSH)	BNCT Carrier	Passive diffusion/EPR effect (in nanocarriers).	Very high boron content per molecule ($12/times $ B).	Poor intracellular uptake; lack of active targeting mechanism without modification.	[48,58]
Carboranes	Drug Design (Pharmacophores)	Hydrophobic moiety interacting with receptor pockets.	Increases metabolic stability; high lipophilicity (passes BBB).	Extreme hydrophobicity can lead to solubility issues and nonspecific binding.	[55,56,57]

**Table 5:** Comparative overview of boron detection and imaging methods.

Method	Principle	Sensitivity/Detection Limit	Invasiveness	Main Limitations	References
ICP-MS	Mass spectrometry of ionized sample	Very High (ppt to ppb range)	Ex vivo (Destructive)	Requires tissue digestion; no spatial information; matrix interferences.	[101,102]
ICP-OES	Optical emission spectroscopy	Moderate (ppb to ppm)	Ex vivo (Destructive)	Lower sensitivity than ICP-MS; requires larger sample volume.	[101]
^11^B MRI	Magnetic resonance of boron nucleus	Low (~mM range)	In vivo (Non-invasive)	Short T_2_ relaxation time; requires very high boron concentrations for signal.	[103,115]
PET	Positron emission (e.g., ^18^F-BPA)	High (pM to nM)	In vivo (Non-invasive)	Indirect measurement (detects label, not boron); requires radiolabeling facilities.	[112,113]
SIMS	Secondary ion mass spectrometry	High (subcellular)	Ex vivo (Tissue sections)	Expensive; complex sample preparation (freezing/drying); vacuum required.	[118,120]
Neutron Capture Radiography	Detection of α-tracks on sensitive films	High	Ex vivo (Tissue sections)	Time-consuming; requires neutron source; no longer standard for rapid screening.	[115,116]

## Data Availability

No new data were created or analyzed in this study.
